# Aspirin administration might accelerate the subsidence of periprosthetic joint infection

**DOI:** 10.1038/s41598-020-72731-y

**Published:** 2020-09-29

**Authors:** Yi Ping Wei, Ju Chun Chien, Wei Hsin Hsiang, Shan Wei Yang, Chun Yu Chen

**Affiliations:** 1 Department of Orthopaedics, Kaohsiung Veteran General Hospital, 386, Ta-Chung 1st Rd, Kaohsiung, Taiwan, ROC; 2 Department of Radiation Oncology, Kaohsiung Veteran General Hospital, Kaohsiung, Taiwan, ROC; 3 Departement of Pharmacy, Tri-Service General Hospital Penghu Branch, Penghu, Taiwan, ROC; 4 Department of Occupational Therapy, Shu-Zen Junior College of Medicine and Management, Kaohsiung, Taiwan, ROC

**Keywords:** Infectious diseases, Bacterial infection

## Abstract

Since the past decade, aspirin, a popular anti-inflammatory drug, has been increasingly studied for its potential antimicrobial and antibiofilm activity with promising results, but studies were limited to in vitro and in vivo investigations. Moreover, evidence concerning the beneficial effects of aspirin on the treatment of biofilm-related infections in real-world population is limited. Thus, this study aimed to investigate whether aspirin could promote infection control for patients with periprosthetic joint infections (PJIs). A single-center database was searched. Regular aspirin exposure was defined as a prescription of aspirin for > 6 months before diagnosis of PJIs and consecutive use during the PJI treatment course at a dose ≧ 100 mg/day. General data, treatment modalities, and recurrence status were collected from medical records by an independent orthopedic surgeon. From January 01, 2010, to February 17, 2019, 88 patients who met the PJI criteria were identified and included in this study. Of these patients, 12 were taking aspirin regularly during the infectious events. In the Cox proportional hazards model, multivariate analysis revealed that the aspirin group demonstrated significant benefit via superior resolution of PJIs (HR 2.200; 95% CI 1.018–4.757; *p* = 0.045). In this study, aspirin is beneficial for infection resolution when combined with the current standard of PJI treatment and conventional antibiotics in the management of PJIs.

## Introduction

Periprosthetic joint infection (PJI) is a condition marked by progressive inflammation in the joint after arthroplasty. In PJI, both bones and implants can be infected with biofilm development which could last for a long time. Since the joint cavity has no blood vessel, antibiotics can barely reach the infected area through the bloodstream, limiting its therapeutic effect. Studies reported that approximately 60% of PJIs are caused by *Staphylococcus aureus*, which is difficult to treat because of its various mechanisms in promoting immune evasion, including the formation of a biofilm and persistence in necrotic bone^1–3^. Therefore, recurrent PJI is a great challenge and may be complicated with chronic osteomyelitis or amputation in severe situations. These complications are mentally and financially devastating, causing severe morbidity, prolonged hospitalization, increase in medical cost, and death, in certain cases^1^.

PJIs occur in 1–4%^2^ of total knee arthroplasty (TKA) cases and < 1% of shoulder and hip replacement cases^4,5^. Historically, patient-related risk factors for PJI were as follows: diabetes with poor glycemic control^4^, obesity (body mass index [BMI] > 40 kg/m^2^)^4^, and impaired immune systems (resulting from long-term steroid use or immunodeficiency disorders such as acquired immunodeficiency syndrome [AIDS])^4^. These high-risk patients are found susceptible to PJI complications, such as chronic osteomyelitis or amputation^4^. In previous studies, significant intrinsic risk factors for PJI treatment failure include [BMI] > 40 kg/m^2^, smoking habit, and *S. aureus* infection^6,7^.

Currently, PJI treatment strategies largely rely on a level II evidence of a prognostic study from 2015^4^, which include surgical removal of the infection foci and prolonged antibiotic treatment against biofilm-producing microorganisms. Clinically, debridement with prosthesis retention is the first option for patients with early or late infections with signs and symptoms lasting less than 3 weeks^4^. Patients with loosening prosthesis, unfavorable skin and subcutaneous tissue (skin fistulae) conditions, or unsuccessful debridement procedure, need to undergo two-stage revision surgery; however, some surgeons successfully performed one-stage revision surgery^5^. Ricardo et al.^4^ defined the infection cure same as our definition, and they disclosed the difficulty of treating PJIs when only suppressive antibiotics were used and only 75% of patients who underwent debridement and retention of prosthesis were successfully cured. In a systemic review published in 2004, the success rate of PJI treatment ranged from 14 to 75%^5^. However, a clinical study about time duration from PJI diagnosis to complete treatment has not been carried out.

Several classifications and staging systems are applied to determine the treatment and largely consider the time interval between primary surgery and appearance of signs and symptoms. The most widely used classification was proposed by Zimmerli et al.^5^; cases were differentiated as early (developed earlier than 3 months postoperatively), intermediate (3–24 months) or late (after 24 months) infections^4,5^.

Since the past decade, aspirin (ASA), a popular antipyretic, anti-inflammatory, and analgesic drug and the most commonly used non-steroidal anti-inflammatory drug, has been increasingly studied for its potential antimicrobial and antibiofilm activity with promising results; however, studies were limited to in vitro and in vivo investigations^8–11^. Recently, a possible mechanism of ASA in infection control has been found. Cai et al.^8^ showed that ASA enhanced the antibiotic-induced cell death of *S. aureus* and methicillin-resistant *S. aureus* (MRSA). Zhou et al.^9^ also reported that ASA promoted the effectiveness of antibiotics against biofilm-associated infections. Sedlacek et al.^10^ showed that ASA treatment significantly reduced the risk of *S. aureus* infection in patients undergoing tunnel-catheter dialysis. It is believed that ASA can be a promising novel antibiofilm agent for treating biofilm-related infections. However, evidence concerning the beneficial effects of ASA in clinical infection treatment is limited.

With the above background, this study hypothesized that ASA may have beneficial effects on PJIs. This study was therefore carried out to examine the effects of ASA on PJIs.

## Material and methods

### Data source and study subjects

After receiving approval from the research ethics board of Kaohsiung Veteran General Hospital, Kaohsiung, Taiwan (IRB number: VGHKS19-CT3-03; date of approval: February 18, 2019), a single-center database was searched for patients diagnosed with PJIs on knee or hip joints. Informed consent was obtained from all subjects or their legal guardians. The inclusion criteria were as follows: (1) had undergone primary TKA, primary total hip arthroplasty, or primary hip bipolar hemiarthroplasty at our hospital; (2) was diagnosed with PJI between January 01, 2010, and February, 17, 2019; and (3) met the PJI criteria stated as below by Parvizi et al.^12^ Patients with follow-up < 1 year after the diagnosis of PJI or confirmed to have multiple infections other than PJI in the same hospitalization period were excluded from the study. Patients with impaired immune systems (resulting from long-term steroid use or immunodeficiency disorders such as AIDS) were also excluded.

All medical records were reviewed and summarized by an independent orthopedist to collect the demographic data, clinical and surgical signs and symptoms, laboratory results, and outcomes. The use of patient data from the database was performed in accordance with relevant guidelines and regulations. The status of ASA usage was obtained from the National Health Insurance database (ASA is a prescription drug in Taiwan, so the status of ASA use can be traced in National Health Insurance Pharma Cloud System). Regular ASA exposure was defined as a prescription of ASA for > 6 months before PJI diagnosis and consecutive use during the PJI treatment course at a dose ≧ 100 mg/day.

### Criteria of PJI diagnosis and infection resolution: how person-time was calculated?

The PJI events were assessed based on the combination of the patients’ serum inflammatory markers (C-reactive protein [CRP] and erythrocyte sedimentation rate [ESR]) and diagnostic joint aspiration. PJI was diagnosed according to the definition reported by Parvizi et al.^12^. Patients were considered to have PJI if three of the four following criteria (two suggestive criteria and two confirmatory criteria) were met: (1) ESR > 30 mm/h, (2) serum CRP > 1 mg/dL, (3) synovial white blood cell count > 1760 cells/μL, and (4) synovial PMN > 76%. Cement erosion or peeling off hydroxyapatite-coated prosthesis (loosening prosthesis) may predispose patients to a contiguous infection. Hematogenous seeding (bacteremia and the same causative microorganism in blood as in PJI) and loosening prosthesis shown on radiographs were also recorded to evaluate the severity of PJIs.

For resolution of PJI, the infection was considered controlled in the absence of symptoms and signs of joint infection, CRP < 2 mg/L, or ESR < 20 mm/h^4^. Unsuccessful outcomes were defined as infection persistence and need for long-term oral antibiotics treatment, or if an above-knee amputation (AKA) was subsequently performed.

### Study endpoint and statistics

The primary study endpoint was time to resolution of PJIs. Statistical analyses were conducted using SPSS (version 20, SPSS Inc., Chicago, IL, USA). Fisher exact test was performed to evaluate the distribution of patients’ factors between ASA and non-ASA groups. The association between ASA use and time-to-PJI resolution was evaluated by Cox hazard analysis and Kaplan–Meier survival curve. Other factors including gender, age, smoking habit, underlying diseases (including diabetes mellitus, obesity, and [BMI] > 40 kg/m^2^), and other confounders (prosthesis loosening, hematogenous seeding, *S. aureus* infection, and affected joint) were corrected using Cox hazard regression analysis. We used 40 kg/m^2^ as BMI cut-off according to previous studies discussing risk factors of PJIs^4,13^ and 69 years as age cut-off based on the mean age in the present study (mean age of the total population: 68.8 years).

Significant risk factors for PJI treatment failure in previous studies^6,7^ and factors with *p* value < 0.2 in the univariate analysis^14^ would be included in the multivariate analysis. The final significance level was set at *p* < 0.05.

## Results

Between January 01, 2010, and February, 17, 2019, 101 consecutive patients diagnosed with PJI of the knee or hip joints in our hospital were identified. Thirteen patients (2 had urosepsis, 2 had pneumonia, 3 had multiple organ failure with sepsis, 4 had rheumatoid arthritis, 1 had Cushing disease, and 1 with AIDS diagnosed for 10 years) were excluded due to multiple infection foci at the same clinical course or having impaired an immune system. Overall, 88 patients were included and evaluated (female, 47; male, 41). There were no missing data among our 88 patients. The median follow-up time (from initial confirmation of infection) was 3.0 (interquartile range [IQR] 2.0–5.0) years. Moreover, 41 patients underwent TKA, 28 underwent total hip arthroplasty, and 19 underwent hip bipolar hemiarthroplasty.

According to the classification published by Zimmerli et al.^5^, 81 patients were categorized as having late-onset PJI (occurred > 24 months after prosthesis implantation). Seven patients were categorized as having early-onset PJI (occurred < 3 months after the prosthesis implantation).

All patients were treated according to the same service protocol by four orthopedic surgeons, based on the algorithm published by Zimmerli et al.^5^ as mentioned in the “Discussion.” For the surgical treatment of PJIs, 75 (85.2%) patients underwent two-stage revision surgeries, 2 (2.3%) patients underwent one-stage revision surgery, and 11 (12.5%) patients underwent debridement with prosthesis retention. Intravenous antibiotic therapy was prescribed for all patients, followed by continuous oral antibiotics, and a total antibiotics course continued for at least 6 weeks. Antibiotics were selected according to the antimicrobial susceptibility testing from the serum or wound culture. In cases with a negative culture result, oxacillin was administered.

Among the 88 study patients, 25 showed treatment failure (3 in the ASA group and 22 in the non-ASA group) and 2 of 25 underwent AKA (both of them were in the non-ASA group). The time interval between PJI diagnosis and infection resolution ranged from 1 to 36 months, with a median of 6.00 (IQR, 3.00–12.00) months (Table 1).

**Table 1:** Baseline characteristics of the studied population.

Characteristics	ASA group (N = 12)	Non-ASA group (N = 76)	*p* value^a^
Mean age	77.67 (range 59–88)	67.37 (range 36–88)	
Gender	5 male; 7 female	36 male; 40 female	0.480
Diabetes mellitus	5	36	0.480
Primary arthroplasty	6 total knee arthroplasty, 3 total hip arthroplasty, 3 hip bipolar hemiarthroplasty	35 total knee arthroplasty, 25 total hip arthroplasty, 16 hip bipolar hemiarthroplasty	–
Smoking habit	1	16	0.274
Obesity ([BMI] > 40 kg/m^2^)	0	9	0.249
Late-onset PJI	12	69	0.344
*S. aureus* infection	6	24	0.177
Hematogenous seeding	0	9	0.249
Prosthesis loosening	2	22	0.306
Treatment success	9	54	0.540
Time to infection resolution	median of 2.50 months (IQR, 1.00–9.25 months)	median of 6.00 (IQR, 3.00–12.00) months	–

*IQR* Interquartile range.

^a^ Fisher exact test was performed to evaluate the distribution of patients’ factors between the ASA and non-ASA groups.

Based on the medical records, 12 of the 88 patients had regular ASA use before and during the PJI events, all at a dose of 100 mg/day (9 patients took ASCOTYL cap, 1 took BOKEY cap, and 2 took TAPAL tablets) for previous cardiovascular disease (specifically, 1 patient had valvular heart disease, 4 had received cardiac stenting procedure, 3 had angina pectoris, and 4 had cardiac arrhythmia). The median time for ASA intake was 3.0 (IQR 1.25–4.0) years.

Among the 12 patients in the ASA group, adverse effects associated with ASA were reported in 3 patients, having ASA-related peptic ulcer. No other severe or lethal side effect was reported.

**Figure 1:**
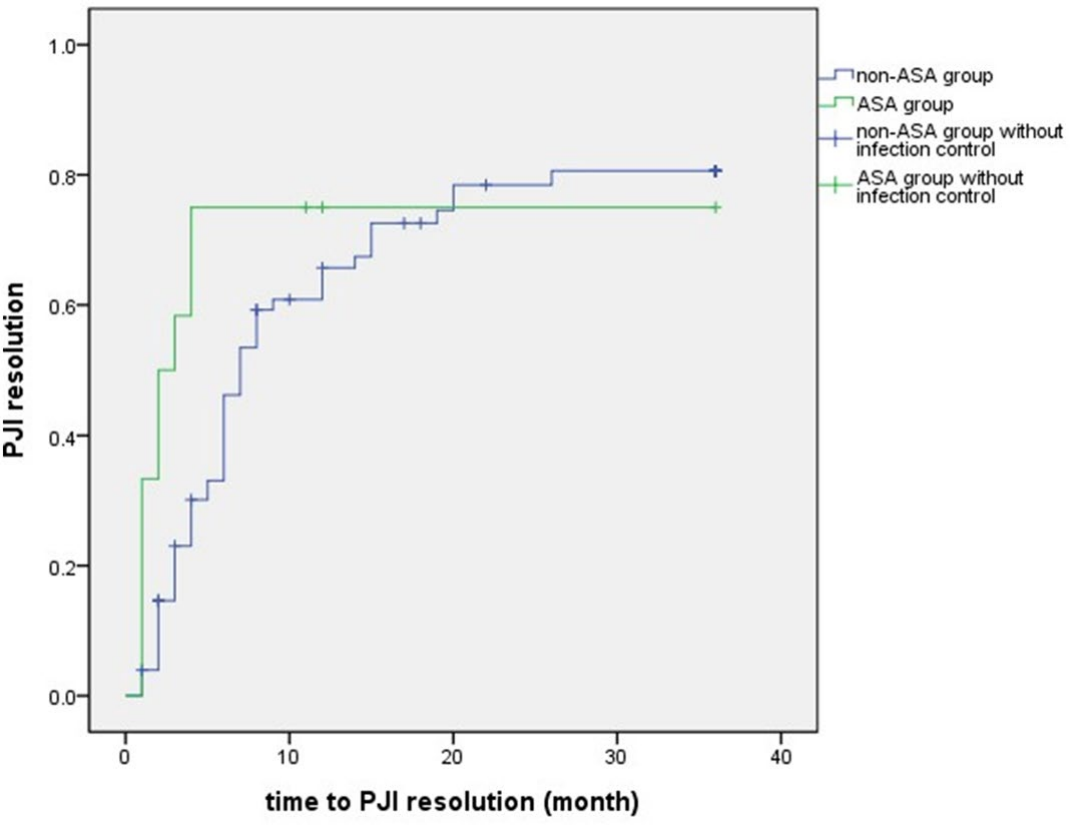
Kaplan–Meier curve of time-to-PJI resolution between the ASA and non-ASA groups. (Blue line, non-ASA group; green line, ASA group; blue line with cross point, case in the non-ASA group without infection control; green line with cross point, case in the ASA group without infection control).

Analysis results of time-to-infection control between the two subgroups are shown in Table 2. In this study, the infection resolution time was not significantly influenced by old age (≥ 69 years) (HR 0.829, 95% CI 0.502–1.369, *p* = 0.465), gender (HR 0.932, 95% CI 0.567–1.531, *p* = 0.781), smoking habit (HR 1.382, 95% CI 0.772–2.474, *p* = 0.277), comorbidities (diabetes mellitus, HR 0.883, 95% CI 0.538–1.451, *p* = 0.623; BMI > 40 kg/m^2^, HR 0.979, 95% CI 0.421–2.278, *p* = 0.961), severity of PJIs (prosthesis loosening, HR 0.721, 95%CI 0.398–1.308, *p* = 0.282; hematogenous seeding, HR 1.579, 95% CI 0.742–3.360, *p* = 0.236; late-onset PJI, HR 0.833, 95% CI 0.359–1.933, *p* = 0.670; *S. aureus* infection, HR 0.918, 95%CI 0.539–1.562, *p* = 0.751), or affected joints (HR 1.116, 95% CI 0.679–1.835, *p* = 0.665). Multivariate analysis was conducted to correct risk factors mentioned in previous studies^5–8^ which significant negative effect in PJI treatment ([BMI] > 40 kg/m^2^, smoking habit, and *S. aureus* infection) and factors with *p* value < 0.2 in the univariate analysis (ASA use). In the multivariate analysis, a trend with benefit was found toward superior resolution of PJIs in the ASA group (HR 2.200; 95% CI 1.018–4.757; *p* = 0.045). The Kaplan–Meier curve of time-to-PJI resolution, stratified by ASA use, is shown in Fig. 1.

**Table 2:** Cox proportional hazard model analysis of time-to-infection resolution.

Risk factor	Univariate analysis		Multivariate analysis	
	Hazard ratio (95% CI)	*p* value	Hazard ratio (95% CI)	*p* value
Gender (male)	0.932 (0.567–1.531)	0.781	–	–
Age (≥ 69 years)	0.829 (0.502–1.369)	0.465	–	–
Smoking habit	1.382 (0.772–2.474)	0.277	1.562 (0.858–2.845)	0.144
Prosthesis loosening	0.721 (0.398–1.308)	0.282	–	–
Hematogenous seeding	1.579 (0.742–3.36)	0.236	–	–
ASA use	1.789 (0.880–3.637)	0.108	2.200 (1.018–4.757)	0.045
Obesity ([BMI] > 40 kg/m^2^)	0.979 (0.421–2.278)	0.961	1.173 (0.492–2.798)	0.719
Late-onset PJI	0.833 (0.359–1.933)	0.670	–	–
Diabetes mellitus	0.883 (0.538–1.451)	0.623	–	–
Affected joint (Knee joint)	1.116 (0.679–1.835)	0.665	–	–
*S. aureus* infection	0.918 (0.539–1.562)	0.751	0.762 (0.432–1.345)	0.349

## Discussion

To the best our knowledge, this study was the first reported clinical research that demonstrated a possible beneficial effect of ASA on PJIs. This finding may help guide the treatment of patients who have an underlying cardiovascular disease and sustained PJIs, especially the older population.

In this study, we did not have a higher treatment success rate in the ASA group (75%) than the previous study (14–75%)^4,5^. We objectively evaluated the time-to-infection-free duration (by serum CRP and ESR level) between the ASA group and non-ASA group and found a trend with benefit on infection resolution in the ASA group (*p* = 0.045). Infection control was not significantly influenced by the presence of confounders (gender, age, DM, smoking habit, obesity, hematogenous seeding, late-onset PJI, *S. aureus* infection, prosthesis loosening, and affected joint).

Future study with greater sample of PJI patients receiving long-term ASA might help further evaluate the role of ASA as a promising novel antibiofilm agent in PJI treatment. Historically, the antibiofilm characteristic of ASA has only been shown in a few in vitro and in vivo studies, i.e., in *Candida albicans*^9,15,16^, *C. parapsilosis*^9^, and *S. xylosus*^17^. Zhou et al.^9^ reported that ASA showed the greatest effectiveness on inhibiting biofilm formation. The results of this study presented that ASA dramatically decreases biofilm formation in *C. albicans*^9^. Moreover, ASA concentration, between 50 and 200 μM, within the range frequently achieved by therapeutic ASA dose in humans, produces a significant level of antibiofilm activity in vitro*.* ASA has been shown to possess potent antibiofilm activity in vitro and could be useful when combined with conventional antibiotics agents in the management of some biofilm-associated infections^9,16^. Al-Bakri et al.^18^ disclosed that ASA was beneficial in eradicating biofilms of *Pseudomonas aeruginosa, Escherichia coli,* and *C. albicans* established on abiotic surfaces. ASA is related with decreasing biofilm formation, consistent with its altering biofilm microarchitecture^18^. *Yi *Jiang et al.^11^ showed that ASA reduced osteolysis and periosteal reaction, inhibited the activation of osteoclasts, promoted the activation of osteoblasts, and facilitated infection resolution in an animal study investigating the strain ATCC43300 of *S. aureus*.

The benefit of ASA for PJIs may be attributed to several factors. In *S. aureus* and MRSA infection, PGE_2_ may accelerate biofilm formation. A study reported that ASA can inhibit the formation of prostaglandin, which can be a virulence factor in biofilm-related infections^16^. Furthermore, the effect of ASA on platelets may be of benefit. The mechanism of disrupting biofilm is similar to the way that ASA interferes with platelet aggregation. Aside from direct antimicrobial effects, aspirin may act on biofilms by interfering in the interaction between *S. aureus* and platelets^19^. In infective endocarditis, a biofilm-related disease, platelets immobilized on the surface of the injured heart valves mediate the adherence of *S. aureus*, a necessary step in the formation of bacterial vegetations.

In addition, ASA has a documented lower incidence of bleeding complications, which may help explain the observed decreased rate of PJI occurrence in the previous study^19^.

In a review of more than 2400 patients with hip or knee arthroplasty infections, gram-positive cocci were found in the majority of hip and knee PJIs^16,20^. Tande et al.^20^ investigated the role of biofilms in PJIs and showed that in several *Staphylococci* species, polysaccharide intercellular adhesion encoded by the *ica* genes contributes to biofilm extracellular matrix.

*S. aureus* is an important pathogen resulting from its virulence and frequency. In addition to being a leading cause of PJIs, it is one of the common pathogens of invasive infection, including nosocomial and health care-associated bloodstream infections, which can lead to PJIs^16,20^.

*S.epidermidis* is the most frequently identified member of coagulase-negative Staphylococci (CoNS). This species causes PJIs primarily through its ability to adhere to prosthesis and produce biofilms. Other species that have been reported to cause PJIs include *S. simulans*, *S. caprae*, and *S. lugdunensis*^20^.

The frequency of culture-negative PJIs varies from 5 to 35%^20^, corresponding with our study (17/88; 19.32%). Culture-negative PJIs is typically of delayed or late onset in a reported study^20^.

For patient with PJIs, surgical intervention is always necessary, and options included debridement with retention of the prosthesis, one- or two-stage prosthesis exchange, resection arthroplasty, arthrodesis, and amputation^4,5^. Debridement with retention is a reasonable choice for those with an early stage (signs and symptoms appear < 3 weeks postoperatively) or acute hematogenous infection. It is indicated for patients with healthy soft tissue and an effective antibiotics against microorganisms^4,5^. Most importantly, patients with loosening prosthesis are not suitable candidates for prosthesis retention.

Intravenous treatment should be administered for 2–4 weeks, followed by continuous oral antibiotics. A total treatment duration of 6 weeks has been suggested in patients with knee or hip prostheses^4,5^.

One-stage revision includes implant removal, debridement, and reimplantation of a new prosthesis during single procedure. It is indicated for patients with only slightly damage in systemic condition (no sign of sepsis) and the absence of difficult-to-treat bacteria (oxacillin-resistant *S. aureus*, *Enteroccocus* species, and fungus)^5^. It has been suggested that the one-stage revision is favorable if the pathogen is known preoperatively and antibiotics treatment is given for at least 2 weeks before the procedure.

In a two-stage exchange, reimplantation of the new prosthesis is delayed for a period of time. It is appropriate for patients with moderate to severe tissue damage (sinus tract on the skin, compromised soft tissue, loosening prosthesis, and sign of sepsis) and the infection of difficult-to-treat microorganisms^5,12^. Moreover, patients with delayed or late onset infection (those that develop 3–24 or >24 months postoperatively) usually present with subtle signs, such as implant loosening^5^. Thus, patients with delayed or late PJI infection should be managed carefully.

Resection arthroplasty consists of debridement and permanent prosthesis removal. An antibiotic-impregnated cemented spacer can keep the limb at the correct length. However, the cemented spacer can be painful to the patients. It is indicated for bedridden patients or patients who are unable to undergo further procedure^5^.

This study has several limitations. First, the small number of patients enrolled into the study limited the treatment success rate and statistical analysis of the time of ASA intake. Second, we could not measure the joint and serum level of ASA and compare the concentrations achieved in the joint fluid. Third, different prosthesis types and different infection stages may also affect the treatment outcomes and treatment strategies. Further studies are therefore needed.

## Conclusion

With the representative study population reflecting the real-world PJI patients, our study revealed that ASA is beneficial for infection resolution when combined with current standards of PJI treatment and conventional antibiotics agents in the management of PJIs in the knee and hip joints.

## Data Availability

The datasets generated during and/or analysed during the current study are available from the corresponding author on reasonable request.

## Abbreviations

AKA: Above-knee amputation
ASA: Aspirin
BMI: Body mass index
CoNS: Coagulase-negative Staphylococci
PJI: Periprosthetic joint infection
TKA: Total knee arthroplasty
IQR: Interquartile range
